# The Design and Analysis of Seroefficacy Studies for Typhoid Conjugate Vaccines

**DOI:** 10.1093/cid/ciy1119

**Published:** 2019-03-07

**Authors:** Xinxue Liu, Virginia E Pitzer, Andrew J Pollard, Merryn Voysey

**Affiliations:** 1 Oxford Vaccine Group, Department of Paediatrics, University of Oxford, United Kingdom; 2 Department of Epidemiology of Microbial Diseases, Yale School of Public Health, New Haven, Connecticut

**Keywords:** typhoid, seroefficacy, typhoid conjugate vaccine, antibody

## Abstract

**Background:**

Demonstrating the efficacy of new Vi-conjugate typhoid vaccines is challenging, due to the cost of field trials requiring tens of thousands of participants. New trial designs that use serologically defined typhoid infections (seroefficacy trials) rather than blood culture positivity as a study endpoint may be useful to assess efficacy using small trials.

**Methods:**

We developed a model for Vi–immunoglobin G antibody responses to a Vi-vaccine, incorporating decay over time and natural boosting due to endemic exposures. From this, we simulated clinical trials in which 2 blood samples were taken during follow-up and the relative risk of a serologically defined typhoid infection (seroefficacy) was computed. We aimed to determine (1) whether seroefficacy trial designs could substantially reduce sample sizes, compared with trials using blood culture–confirmed cases; (3) whether the rate of case detection was higher in seroefficacy trials; and (3) the optimal timing of sample collection.

**Results:**

The majority (>90%) of blood culture–positive typhoid cases remain unobserved in surveillance studies. In contrast, under-detection in simulated seroefficacy trials of equivalent vaccines was as little as 26%, and estimates of the relative risk of typhoid infection were unbiased. For simulated trials of non-equivalent vaccines, relative risks were slightly inflated by at least 5%, depending on the sample collection times. Seroefficacy trials required as few as 460 participants per arm, compared with 10 000 per arm for trials using blood culture–confirmed cases.

**Conclusions:**

Seroefficacy trials can establish the efficacy of new conjugate vaccines using small trials that enroll hundreds rather than thousands of participants, and without the need for resource-intensive typhoid fever surveillance programs.

The recent World Health Organization prequalification and recommendation for use of the typhoid Vi-polysaccharide tetanus-toxoid conjugate vaccine (Vi-TT), has led to renewed hope for the widespread control of typhoid fever in low- and middle-income countries [1, 2]. Vaccine immunogenicity has been shown in a randomized trial in India [3], and the efficacy of Vi-TT was demonstrated in a controlled human challenge study [4]. Large, double-blind, randomized trials of between 20 000 and 42 000 children are ongoing in Nepal, Bangladesh, and Malawi to assess the vaccine’s effectiveness when administered to children in endemic settings [5]. In addition, the population impact of vaccine introduction is being assessed in Navi Mumbai [6].

Other typhoid conjugate vaccines are at varying stages of development and use. A Vi-conjugate vaccine with a recombinant *Pseudomonas aeruginosa* exotoxin A carrier protein was demonstrated to be effective in field trials [7]; a Vi-diphtheria-toxoid conjugate vaccine was immunogenic in a randomized trial in the Philippines [8]; and a Vi-conjugate vaccine with diphtheria cross-reacting material as carrier protein (Vi-CRM197) was immunogenic in a randomized trial in South Asia [9]. A Vi-TT vaccine with a lower polysaccharide content is also licensed in India [10].

The challenge for manufacturers and developers of new-generation typhoid vaccines is to demonstrate vaccine efficacy. The current gold standard for typhoid diagnoses is the culture of *Salmonella* Typhi from the blood of symptomatic patients. As blood culture–confirmed typhoid incidence is low, the sample size required to confirm efficacy in field trials is very large (typically >20 000). When a licensed and effective vaccine is in widespread use, clinical trials with a placebo control become unethical. Instead, new vaccines can be tested against the standard vaccine in a non-inferiority trial. Such trials aim to demonstrate that the new vaccine is at least as good as the standard vaccine. Sample sizes for non-inferiority trials are generally larger than for placebo-controlled trials, since the difference between the 2 vaccines is expected to be small. Due to the required size, conducting large field efficacy studies can be prohibitively expensive. In addition, there are no established, standardized assays for assessing the functional antibody responses to Vi-containing vaccines [11].

In the absence of functional assays or correlates of protection, alternative methods for demonstrating the protection conferred by new typhoid conjugate vaccines are needed. We previously showed that vaccine efficacy can be computed from immunogenicity data alone, by modelling serologically defined infections and comparing the incidence of these infections between randomized groups in a clinical trial [15]. Immunogenicity trial participants in endemic settings will be naturally exposed to *Salmonella* Typhi during trial follow-up, particularly where the disease incidence is high. The detection of Vi-antibody responses to natural exposure can be used to estimate the incidence of clinical or subclinical infections if blood samples are taken from participants at appropriate times. However, since the timing of an infection is unpredictable, infection events may be missed if the antibody response to exposure is small or if the antibody has waned by the time a blood sample is taken. Whilst a Vi-antibody response can only be induced by exposure to bacteria expressing a Vi-polysaccharide, this may not necessarily be *S*. Typhi, as other bacteria can also express the same polysaccharide. A Vi-containing vaccine will prevent infection by any Vi-expressing bacteria and, in a typhoid-endemic setting, these are most likely to be *S*. Typhi infections.

We explored the design of seroefficacy studies by simulating the antibody response to vaccination and the trajectory of decay over time in a hypothetical population with endemic exposure. We simulated the vaccination of 2 groups in clinical trials of a hypothetical, new Vi-conjugate vaccine to an equivalent, established Vi-conjugate vaccine. The simulated trials incorporated 2 blood samples, taken at varying time points, to measure anti-Vi antibody levels. A rise in antibody levels, or seroincidence, was the primary outcome, representing vaccine failure.

The goal of our simulations was to determine the optimal timing of sample collection when conducting such a study and to assess the degree of under-detection of infections, the magnitude of biases in efficacy estimates, and the impact of the timing of blood samples on vaccine efficacy calculations.

## METHODS

### Population-level Antibody Response to Vaccination and the Trajectory of Decay

We simulated Vi–immunoglobin G (IgG) antibody data (*Ab*) for recipients of a hypothetical Vi-conjugate vaccine. The initial antibody response for each individual (*i*) at time 0 (*t* = 0) was simulated as a log_10_-transformed value from a normal distribution with a geometric mean of 1000 EU/mL, similar to the antibody levels seen in previous immunogenicity studies of Vi-TT [3].

Antibodies induced by vaccination decay more rapidly in the first year post-vaccination, after which decay rates slow and plateau in the absence of exposure to the antigen [16–18]. We simulated this trajectory, using a cubic polynomial function.

$$Ab_{ti}=b_{1i}t+b_{2i}t^{2}+b_{3i}t^{3}$$

Here, *Ab*_*ti*_ is the antibody level at time *t* for individual *i* and *b*_*1i*_*, b*_*2i*_, and *b*_*3i*_ are the coefficients in the cubic polynomial function for individual *i* (see Table 1).

**Table 1.** Model Parameters and Definitions Used in Simulations

Parameter Definition	Symbol	Value(s)	Source
**Serological models**			
Log_10_-transformed Vi-IgG antibody concentration for participant i at time t = 0	*Ab*_*0i*_	Control vaccine ~ N(3.0, 0.33^2^), equivalent test vaccine ~ N(3.0, 0.33^2^), and less immunogenic test vaccine ~ N(2.7, 0.33^2^)	Assumption (equivalent to geometric mean concentrations: 1000 and 500 EU/mL respectively)
Probability of exposure to *Salmonella* Typhi over the 2-year follow-up period	*p*_*exp*_	Low exposure 0.25,medium exposure 0.5, and high exposure 0.8	Assumption
Exposure status	*E*_*i*_	~Bernoulli(*p*_*exp*_)	…
Time at which exposure occurs, in years	*t*_*exp_i*_	~U(0.1, 2.0)	…
Log_10_-transformed Vi-IgG antibody concentration for participant i at time t, if uninfected	*Ab*_*ti*_ = *b*_*1i*_*t* + *b*_*2i*_*t*^*2*^ + *b*_*3i*_*t*^*3*^	*b*_*1i*_ ~ N(2.90,0.10^2^), *b*_*2i*_ ~ N(1.85,0.02^2^), and *b*_*3i*_ ~ N(-0.40,0.01^2^)	See Supplementary files
Probability of infection at time t for participant I, if exposed	*p*_*inf_i*_	$\frac{e^{1.7638-0.9597Ab_{ti}}}{1+e^{1.7638-0.9597Ab_{ti}}}$	^[4]^
Infection status, if exposed, *E*_*i*_ = 1	*D*_*i*_	~Bernoulli(*p*_*inf*_)	…
Log_10_-transformed Vi-IgG antibody generated in response to infection for participant i, if infected	*Ab*_*di*_	*~*N (2.25, 0.396^2^)	Assumption
Total log_10_-transformed Vi-IgG antibody at time of infection for participant i, if infected	*Ab*_*tdi*_	$\log_{10}\left(10^{Abdi}+10^{Abti}\right)$	…
**Clinical Trial Scenarios**			
Time at first blood sample, in years	*S1*	0.5 to 1.9, by 0.1 [N = 15]	…
Time at second blood sample, in years	*S2*	1.0 to 2.0, by 0.1 [N = 11]	…
Total number of scenarios	*Where S1* < *S2*	N = 110	…

*Ab*_*ti*_ is the antibody level at time *t* for individual *i,* and *b*_*1i*_*, b*_*2i*_ and *b*_*3i*_ are the coefficients in the cubic polynomial function for individual *i.*

Abbreviations: *D*_*i*_, an individual’s infection status; *E*_*i*_, an individual’s exposure status; IgG, immunoglobin G; *p*_*exp*_, probability of exposure; N, normal distribution; *p*_*inf*_, probability of infection. S1, first blood sample; S2, second blood sample; U, uniform distribution.

**Figure 1.**
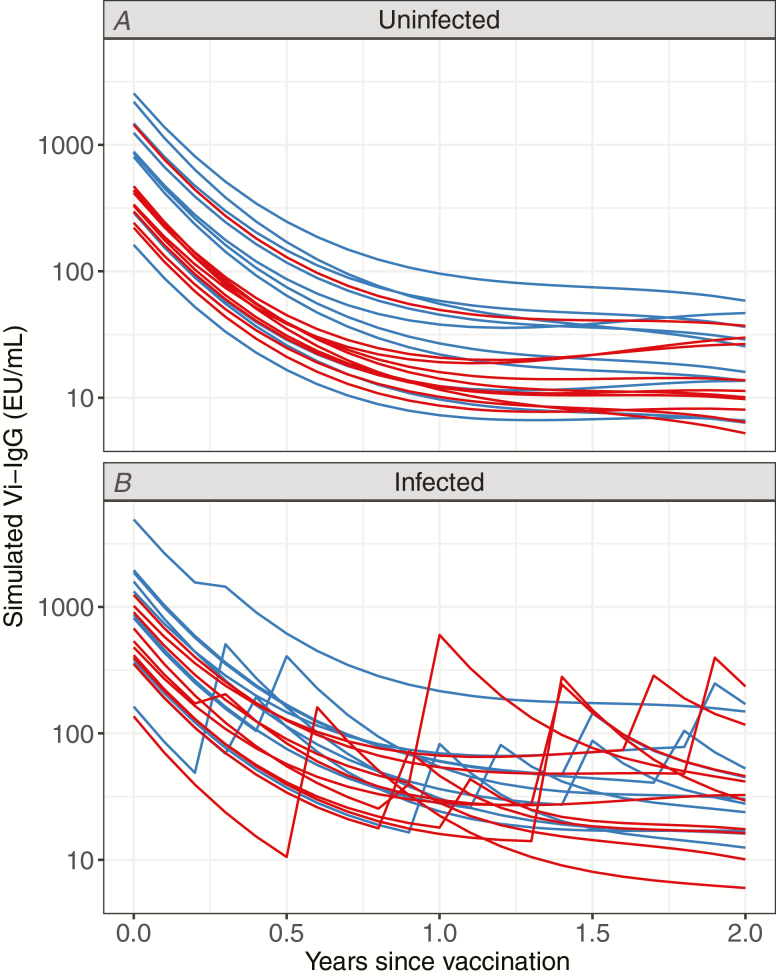
Vi-IgG trajectories for randomly selected (*A*) uninfected and (*B*) infected participants in simulated clinical trials who received 1 of 2 non-equivalent Vi-conjugate vaccines. Each curve represents the simulated data generated for 1 individual. As a large number of individual antibody trajectories were simulated in the models, only a small, random selection are shown, in order to illustrate the data underlying the models presented. The blue lines indicate individuals assigned to the standard vaccine; the red lines indicate those assigned to the less-immunogenic vaccine. Abbreviation: IgG, immunoglobin G.

Figure 1 displays the resulting antibody trajectories for a randomly selected subset of individuals.

### Simulation of Infection Due to Natural Exposure

#### Probability of Infection

We set the probability of exposure (*p*_*exp*_) to be constant for all individuals across the 2-year follow-up period; the time at which exposure occurred was assumed to be uniformly distributed. We chose 3 levels for *p*_*exp*_ (0.8, 0.5, and 0.25), representing the probability that an individual would be exposed to the bacteria at some point during a 2-year follow-up period. The probability of exposure to *S*. Typhi will be higher than both the probability of infection and the probability of symptomatic blood culture–positive typhoid fever. However, the exact nature of the relationship between exposure and disease has not been well characterized, and our exposure probabilities do not necessarily correlate with the high, medium, and low incidences of blood culture–positive disease. The probability of infection (*p*_*inf_i*_) was determined for each participant using a logistic function with parameters derived from a logistic regression of antibody levels in a controlled human infection study [4] (Table 1).

For each individual, exposure status (*E*_*i*_ = 0,1) and infection status (*D*_*i*_ = 0,1) were generated from independent Bernoulli distributions with parameters *p*_*exp*_ and *p*_*inf_i*_, respectively, with the additional condition that if a participant was unexposed (*E*_*i*_ = 0), they remained uninfected (*D*_*i*_ = 0).

#### Antibody Response to Infection

The Vi-IgG response to infection was generated in a similar way to the Vi-IgG response to vaccination. However, we assumed antibody responses to infection were smaller and more variable than vaccine-induced responses, based on data from human challenge studies [19].

Total antibody levels at any time post-infection were the sum of the residual, vaccine-induced antibody and the waning, infection-induced antibody.

All model parameters are displayed in Table 1.

### Calculating Seroefficacy and Seroincidence

We simulated clinical trials, in which the observed efficacy of the vaccine was estimated from the antibody concentrations at 2 blood sampling time points during follow-up. The difference between the log_10_ antibody levels at the 2 time points was computed, and a 2-component Gaussian mixture model was fitted to the differences. Individuals with antibody levels that “sharply” increased or decreased between these 2 time points were presumed to be those who had been infected either after the first blood sample (antibody sharply increasing) or shortly before the first blood sample (antibody sharply decreasing). Rather than classify each individual as infected or not, the posterior probability of being in the second component in the mixture model (those with sharply increasing or decreasing antibody levels) was summed across participants in each randomized vaccine group to give the seroincidence of infection in each group. When comparing 2 groups of participants who received the 2 different vaccines, the ratio of the summed posterior probabilities in the 2 randomized groups was computed to give the relative risk of infection. The full code for these calculations is supplied in the Supplementary Material.

### Simulation of Clinical Trial Scenarios for Testing Equivalent Vaccines

Using the above methods, we simulated the clinical trials of 2 different but equivalent Vi-conjugate vaccines: a control vaccine and a test vaccine. Hypothetical trials, each with 500 participants (250 in each arm), were generated for scenarios in which 2 blood samples were taken at varying times during the follow-up period. The first blood samples were taken between 0.5 and 1.9 years after vaccination; the second samples were taken between 1.0 and 2.0 years into the follow-up period (Table 1). Antibody measures from only these 2 time points were retained in the data set, with the remaining antibody data discarded, to align with the reality that the antibody trajectory for any participant in a clinical trial is unknown, except at the specific times blood samples are taken.

In each simulated clinical trial, the true relative risk of infection was the ratio of the infected proportions in each group:

$${\text{RR}}_{\text{true}}=\frac{{}^{\sum {\text{D}}_{\text{i ​​}\;\text{​​ test}}}\text{​​}╱\text{​}{\text{​}}_{250}\;}{{}^{\sum {\text{D}}_{\text{i ​​}\;\text{​​ control}}}\text{​​}╱\text{​}{\text{​}}_{250}\;}$$

The observed relative risk of infection (

$$RR_{obs}$$),

computed using seroincidence was compared to the true relative risk of infection. The bias was computed for each trial as the ratio of these 2 relative risks:

$$\text{Bias ​​}\;\text{​​ Ratio}=\frac{RR_{obs}}{RR_{true}}$$

### Simulation of Clinical Trial Scenarios for Testing Non-equivalent Vaccines

Simulations were repeated for additional scenarios in which the test vaccine was less immunogenic than the control vaccine. In these scenarios, the initial Vi-IgG response to vaccination was assumed to be lower for the test vaccine than the control vaccine.

## RESULTS

### Under-detection of Infections

**Figure 2.**
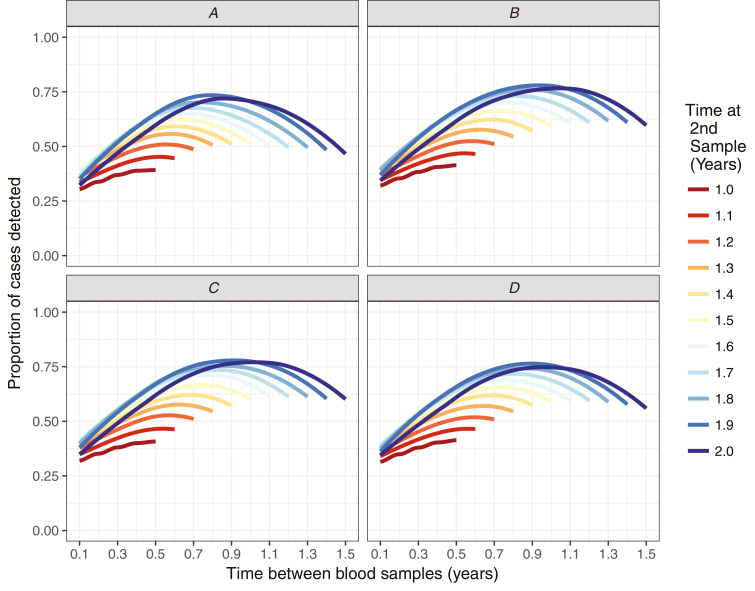
The overall proportion of infected cases detected in simulated trials, using seroincidence as the primary outcome. *A*, Trials of equivalent vaccines. *B*, Trials of non-equivalent vaccines, *C*, Trials of non-equivalent vaccines in low-exposure settings. *D*, Trials of non-equivalent vaccines in high-exposure settings.

Calculating the seroincidence of infection from 2 blood samples during follow-up underestimated the true incidence of infection. The degree of underestimation depended on the timing and spacing of blood samples. Figure 2 shows the degree of under-detection across different scenarios by comparing the seroincidence (proportion of people infected) to the true infection rate. The proportion of cases detected ranged from 0.30 to 0.74. In trials of equivalent vaccines (Figure 2A), under-detection of infections was minimized for scenarios in which the second sample was taken 1.8 to 2.0 years after vaccination and with a gap between samples of 0.6 to 0.9 years.

In trials of non-equivalent vaccines (Figure 2B), under-detection of infections was similar to trials of equivalent vaccines. Under-estimation was minimized for scenarios in which the second sample was taken 1.8 to 2.0 years after vaccination and with a gap between samples of 0.9 to 1.0 years. The degree of underestimation did not differ appreciably for low-exposure or high-exposure settings (Figure 2C and 2D).

### Bias in Relative Risk Estimates

**Figure 3.**
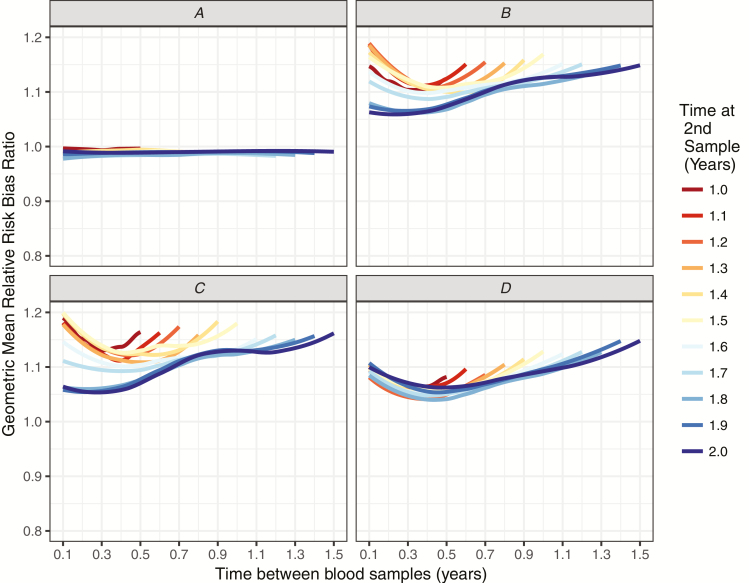
Bias in estimated relative risks in seroincidence studies. *A*, Trials of equivalent vaccines. *B*, Trials of non-equivalent vaccines. *C*, Trials of non-equivalent vaccines in low-exposure settings. *D*, Trials of non-equivalent vaccines in high-exposure settings. A bias ratio of 1.0 represents no bias.

For equivalent vaccines, relative risk estimates from seroefficacy trials were very close to the true relative risks, with bias estimates close to 0.98 in most scenarios (with values of 1.0 representing no bias). Biases did not vary with the blood sampling time points when the vaccines were equally efficacious (Figure 3A).

When clinical trials were simulated for a new vaccine with lower immunogenicity, the relative risks from seroefficacy trials were slightly inflated. Figure 3B shows a scenario in which the new vaccine produced a geometric mean of 500 EU/mL, compared to the standard vaccine, which induced a geometric mean of 1000 EU/mL. Infection was more common with lower antibody levels and, therefore, the simulated true relative risk of infection for these trials was 1.13. The degree to which seroefficacy trials overestimated this relative risk varied according to the timing of blood samples. The relative risks derived from the seroincidence studies of non-equivalent vaccines were between 1.05 and 1.19 times higher than the true relative risk (Figure 3A). The least bias occurred in trials with a second sample taken 1.8 to 2 years after vaccination and with less than 0.5 years between samples. Similar results were seen for trials in the low-exposure settings (Figure 3C). For high-exposure settings, the biases were less overall, with relative risks ranging from 1.04 to 1.16 times the true values (Figure 3D).

### Sample Sizes for Non-inferiority Trials

**Figure 4.**
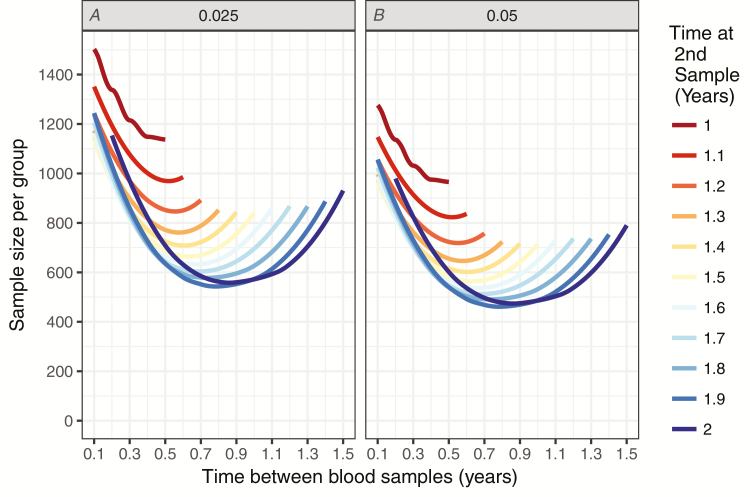
Sample size for non-inferiority test for the relative risk of infection. Margin = 1.5, alpha = 0.025 or 0.05 (*A* and *B*, respectively), power = 0.8. The seroincidence proportions used for power calculations range from 0.08 to 0.197 and are the observed seroincidences from simulations shown in Figures 2A and 3A.

Figure 4 shows the required sample sizes for non-inferiority trials of a new Vi-conjugate vaccine with varying blood sampling time points. Trial designs with the largest proportion of infections detected had the smallest sample sizes.

### Typhoid Seasonality

Simulations incorporating the seasonality of typhoid infections into the models showed that the optimal time to take blood samples was immediately after the typhoid season (Supplementary Material).

## DISCUSSION

Small seroefficacy studies, enrolling hundreds rather than thousands of participants, can be used to assess the non-inferiority of new Vi-conjugate vaccines as compared with similar, licensed vaccines, and can produce unbiased estimates of the relative risks of infection.

Even with established surveillance procedures in place, missed cases of typhoid fever occur when participants with fevers attend clinics that are not part of the study surveillance program, do not seek medical care, or seek medical care but do not consent to blood cultures. Even for those who do consent, the sensitivity of blood cultures is low [20]. Additionally, some infections are asymptomatic and, therefore, not detectable using surveillance, yet contribute to transmission. Vaccine efficacy against asymptomatic infections is of great importance, as the prevention of transmission by an effective vaccine will result in herd protection. The under-detection of blood culture–confirmed typhoid fever in surveillance studies is very high (~90%) [20, 21], and must be incorporated into mathematical models and adjusted for in surveillance studies [22, 23]. In contrast, our models show that under-detection in seroefficacy studies is substantially lower, and can be as low as 25%.

Even with perfect case ascertainment, the incidence of blood culture–positive typhoid fever will be lower than the seroincidence of typhoid infections, as seroefficacy studies include both clinical and subclinical infections. The higher incidences and better detection rates in seroefficacy studies mean that vaccine efficacy can be shown using small numbers of participants, whereas field efficacy studies with blood culture–positive fever as a primary outcome require tens of thousands of participants and rely on complicated disease surveillance programs with variable accuracy levels.

Seroepidemiological surveys are widely used to estimate the incidence of typhoid and other infections in populations, either by comparing antibody levels to a pre-defined threshold, by fitting mathematical models, or by using mixture models [18, 24, 25]. For typhoid, Vi-IgG has been used to estimate the seroprevalence of infections in a population using cut-points of Vi-IgG >64 or >100 EU/mL [21], but such thresholds are non-transferable to studies with different Vi assays.

In clinical trials, serological methods to assess vaccine efficacy are uncommon. We conducted 1 post hoc analysis of a typhoid immunogenicity trial [15] and have analyzed pneumococcal conjugate vaccine data using similar methods [26], but we are not aware of other studies. The modelling of seroincidence based on the change in antibody titers between 2 time points is a method that can be used regardless of the assay, and does not rely upon establishing protective thresholds.

Whilst the number of participants is a key component of the research cost for clinical trials, the length of follow-up also has an important influence on logistics, and is directly affected by the choice of blood sampling times. Our simulations reveal that the choice of blood sampling time points in seroefficacy studies greatly impacts the accuracy of the estimates of disease incidences, as well as the biases in relative risks of infection. A longer follow-up period also has practical implications, with more participants likely to be lost to follow-up. Our simulations show that studies with samples taken in the first year after vaccination, when antibody levels are waning, substantially underestimate the true incidence of infections. In such situations, where participants have been recently vaccinated, the antibody response to infection is difficult to distinguish from the vaccine-induced antibody, resulting in under-detection of infection events. Seroefficacy studies of less than 18 months duration are, therefore, not recommended. We have demonstrated that the best design for a seroefficacy trial of a Vi-conjugate vaccine includes blood samples taken at 14–16 months and 23–24 months after vaccination. These timings minimize the under-detection of infections (from 70% down to 26%) and result in a bias of less than 2% in trials of equivalent vaccines. However, good estimates can still be obtained with a final sample taken 18 months after vaccination and a 6–7 month gap between samples. In such situations, the under-detection of events is 40%, but a further 100 participants per group is required due to the lower detection rate. An assessment of seasonality showed that, when the disease incidence is highly seasonal, samples taken immediately after the season has ended, before antibody levels have had time to decay, provide the highest case detection rates.

Our simulations show that seroefficacy trials produce slightly inflated estimates of the relative risks when vaccines are non-equivalent. In such situations, the protective efficacy of a new, less-immunogenic vaccine is slightly underestimated, leading to the inflation of the relative risk.

Our simulations focus only on scenarios in which the antibody responses to infections are similar with both vaccines. If the priming of the immune system with 2 vaccines is substantially different (such as in a trial of a Vi vaccine vs a meningococcal vaccine), such that the antibody responses to natural exposure are altered with the new vaccine, then seroefficacy studies may not be useful. In such situations, the Vi-antibody response to infection in the control arm will not be similar to that of the Vi-conjugate–primed arm. Other antibodies that are induced by infection, but not by vaccination, such as the recently identified *Salmonella* protein antigens [27], may be a useful alternative. The design of such studies would differ, however, as antibody trajectories would be flat, rather than decaying steeply in the immediate post-vaccination period.

### Limitations

Of necessity, simulation studies are based on assumptions, and the accuracy and validity of these assumptions affects the accuracy of the conclusions. There are limited data on the trajectory of Vi-IgG decay after vaccination with a Vi-conjugate vaccine. We have assumed a polynomial function, as this fits with what is known about Vi antibody levels, as well as antibody responses to other bacterial conjugate vaccines. However, the trajectory of decay may vary in different populations and for different vaccines, and it is unclear what impact this has on the model outputs. Furthermore, we estimated the probability of infection for a given antibody titer based on challenge study data, which may differ from an endemic setting.

### Conclusion

With the prequalification of the first typhoid conjugate vaccine, there is great hope for the protection of millions of children against this serious and sometimes fatal disease. Additional, new conjugate vaccines are essential to increase vaccine production and broaden the resilience of the global supply, which currently relies on only 1 manufacturer. Seroefficacy studies can establish the efficacy of these new vaccines, using small trials that enroll hundreds rather than thousands of participants and that dispense with the need for complicated disease surveillance programs.

## Supplementary Data

Supplementary materials are available at *Clinical Infectious Diseases* online. Consisting of data provided by the authors to benefit the reader, the posted materials are not copyedited and are the sole responsibility of the authors, so questions or comments should be addressed to the corresponding author.

Supplementary Files

## Notes

***Author contributions.*** M. V. and X. L. planned the study and conducted the analysis. M. V. drafted the paper. X. L., A. J. P., and V. E. P. reviewed the paper. All authors reviewed and approved the final version of the manuscript.

***Disclaimer.*** The National Institute for Health Research (NIHR) had no involvement in the design and conduct of the study; analysis and interpretation of the data; preparation, review, or approval of the manuscript; or decision to submit the manuscript for publication. The findings and conclusions contained within are those of the authors and do not necessarily reflect the positions or policies of the Bill & Melinda Gates Foundation. The views expressed in this manuscript are those of the authors, and do not necessarily reflect the views of the Joint Committee on Vaccination and Immunisation, the National Health Service (NHS), the NIHR, the UK Department of Health and Social Care, or the World Health Organization.

***Financial support.*** This publication is based on research funded in part by a grant from the Bill & Melinda Gates Foundation (OPP1151153).

***Supplement sponsorship.*** This supplement is sponsored by the University of Maryland School of Medicine Center for Vaccine Development and Global Health (CVD).

***Potential conflicts of interest.*** A. J. P. chairs the UK Department of Health and Social Care’s Joint Committee on Vaccination and Immunisation; is a member of the World Health Organization’s Strategic Advisory Group of Experts; and has received grants from Okairos, Pfizer, Wellcome Trust, and the National Institute for Health Research (NIHR) Oxford Biomedical Research Centre, outside the submitted work. M. V. received funding from a NIHR Doctoral Research Fellowship (number DRF-2015-08-048), during the conduct of the study. V. E. P. reports grants from the Wellcome Trust (Strategic Award 106158/Z/14/Z), during the course of the study. X. L. reports no potential conflicts. All authors have submitted the ICMJE Form for Disclosure of Potential Conflicts of Interest. Conflicts that the editors consider relevant to the content of the manuscript have been disclosed.
