# Supplementary material for: The Design and Analysis of Seroefficacy Studies for Typhoid Conjugate Vaccines
Source: Clin Infect Dis. 2019 Mar 7;68(Suppl 2):S183–90. doi: 10.1093/cid/ciy1119 (PMC6405265; doi:10.1093/cid/ciy1119)
Supplement: Supplementary Files [file ciy1119_suppl_supplementary_files.docx]

Supplementary Files

# Antibody decay

The trajectory of decay for Vi-IgG antibody used in our modelling was informed by the analysis of the decline in antibody after pneumococcal conjugate vaccination in UK infants aged 13 months to 3 years (unpublished data). Plots of the cubic polynomial function fitted to the anti-pneumococcal data for three indicative serotypes are displayed below.

*Figure S1 Pneumococcal antibody concentrations in children aged 13 to 36 months who received PCV13 booster vaccination at 12 months of age*


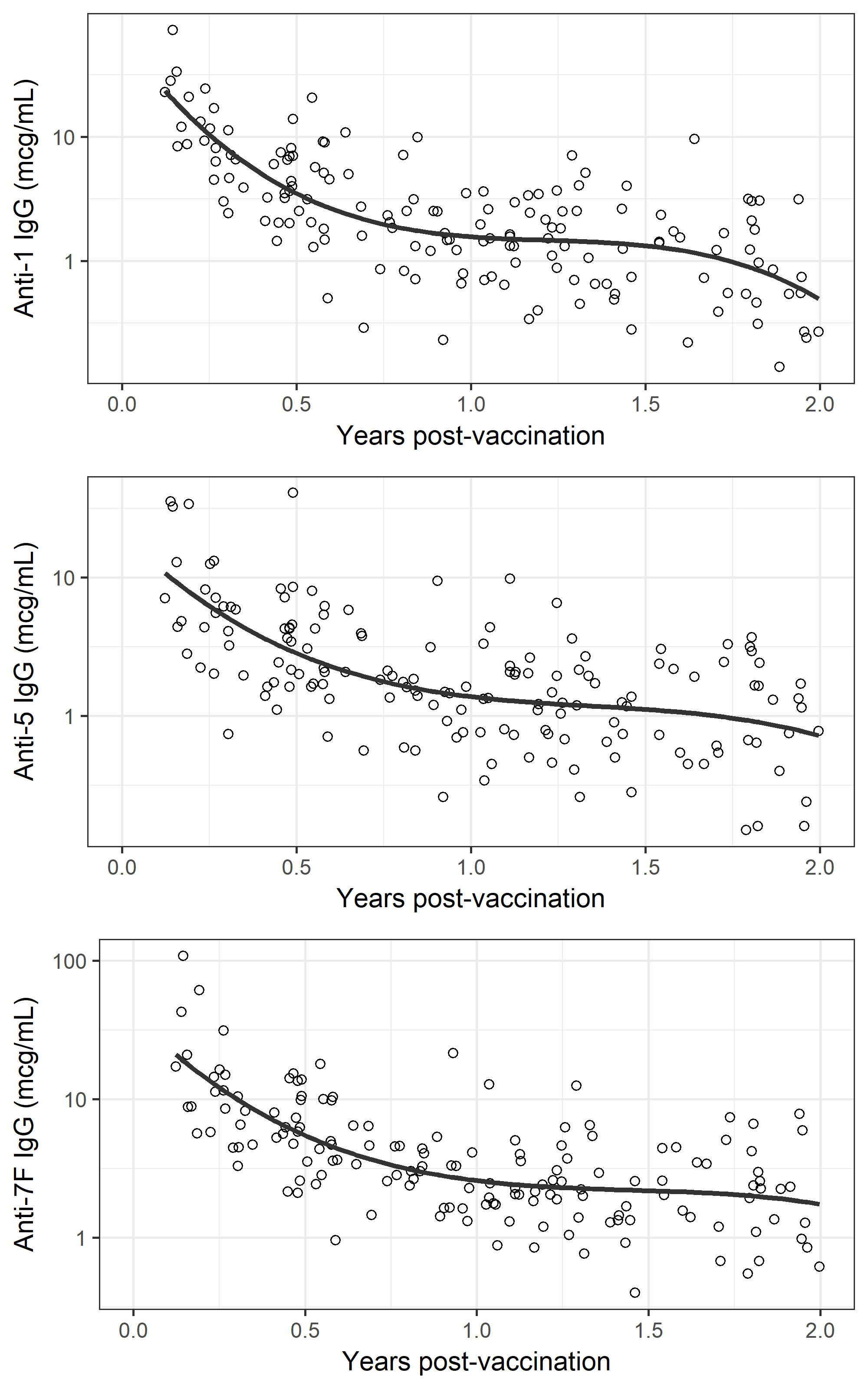


# Exploring Seasonal variation in typhoid incidence and the impact on seroefficacy trials

## Simulation 1

In the main simulations, the probability of exposure was held constant over time. in reality, typhoid fever shows seasonal variation and this may impact on the design of a seroefficacy study, in particular, whether a study starts before the main typhoid season or after it. Additional simulations were conducted to explore the effect of seasonality on the proportion of cases detected in seroefficacy clinical trials.

These simulations follow those for trials of equivalent vaccines in the main paper, but the probability of exposure to S.Typhi follows a distinct seasonal pattern as shown in Figures S2 & S5, rather than being held constant. In the first simulation (Figure S2), participants are enrolled into the study before the typhoid season, and in the second (Figure S5), they are enrolled in the middle of the season. Plots of the proportion of cases detected reveal the distinct seasonality (Figures S3 & S6). In both situations, the highest detection rates occur when blood samples are taken immediately after each typhoid season (Simulation 1: 0.7 years and 1.7 years; Simulation 2: 1.2 years and 2.0 years) as this ensures those individuals infected in the preceding season have antibody levels that would still be high. Bias was unaffected by seasonality for trials of equivalent vaccines (Figures S4 & S7).

Figure S2 Time at which exposure to S.Typhi occurs in simulated seroincidence studies assuming typhoid incidence is seasonal and participants are enrolled before the typhoid season begins


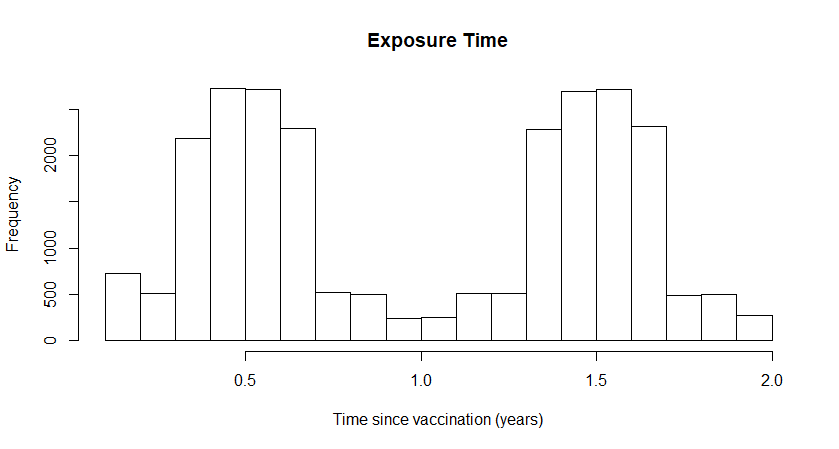


Figure S3 The overall proportion of infected cases detected in simulated trials using seroincidence as the primary outcome


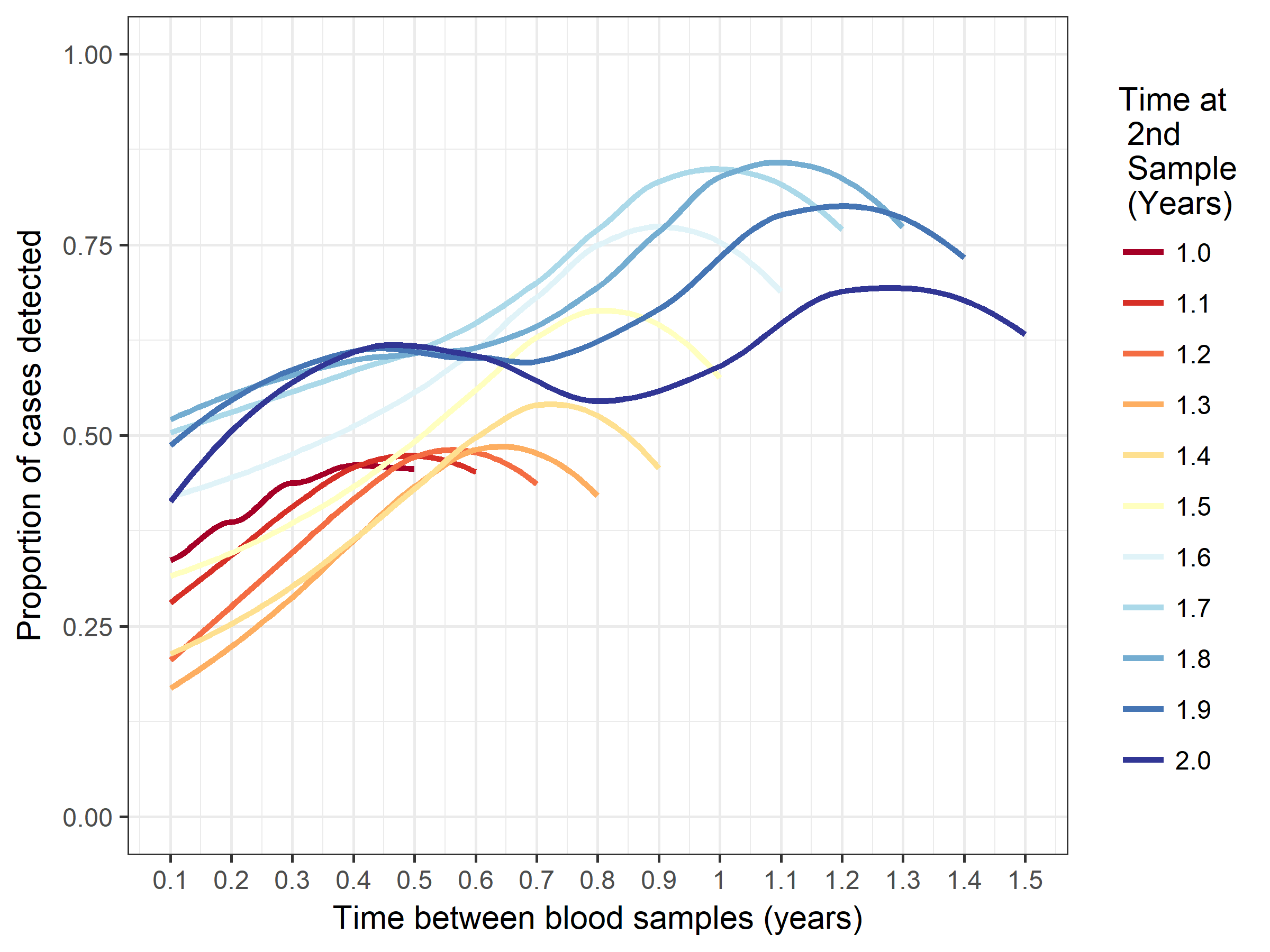


Figure S4 Bias in estimated relative risks in seroincidence studies


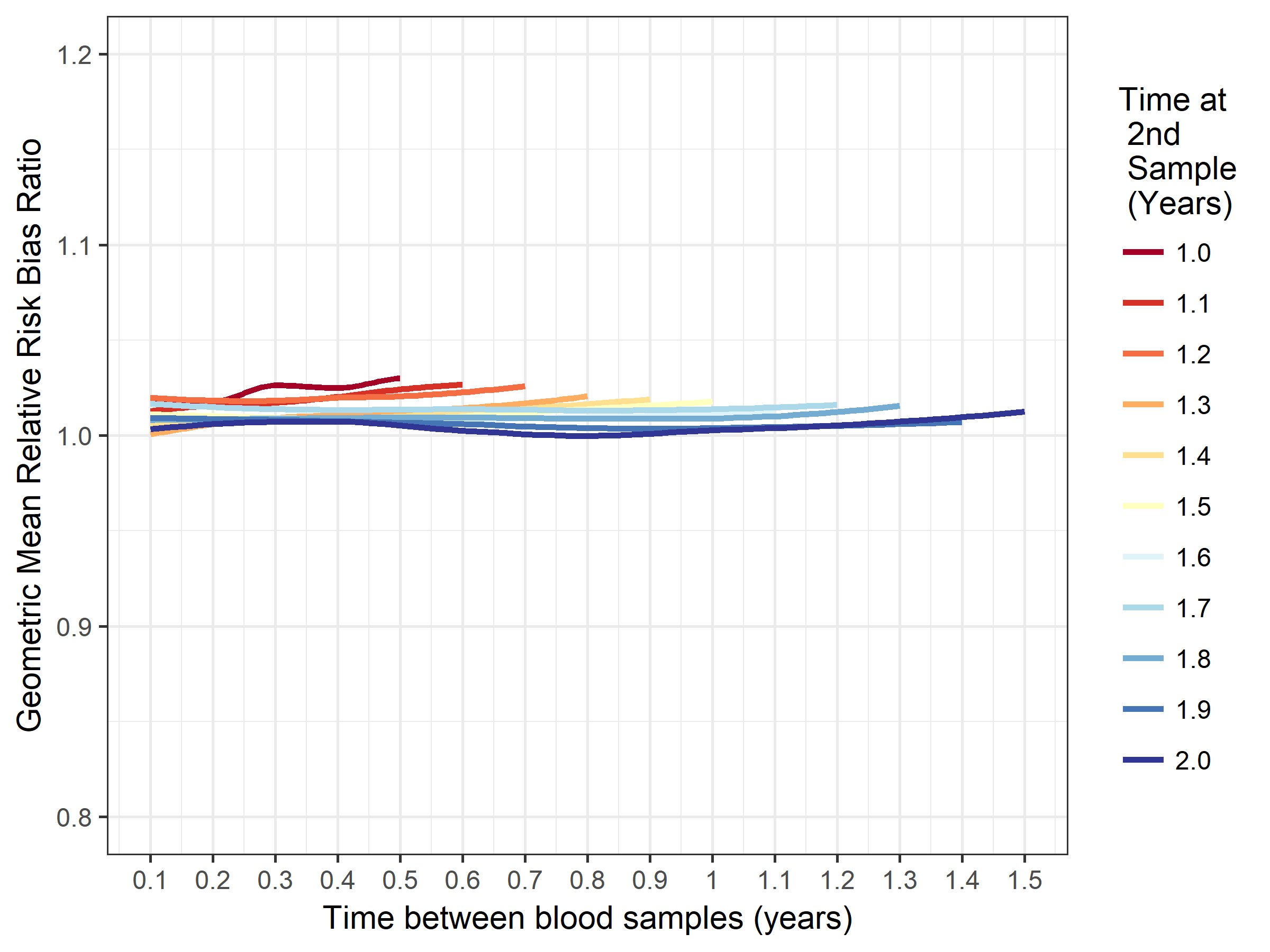


## Simulation 2

Figure S5 Time at which exposure to S.Typhi occurs in simulated seroincidence studies assuming typhoid incidence is seasonal and participants are enrolled in the middle of the typhoid season


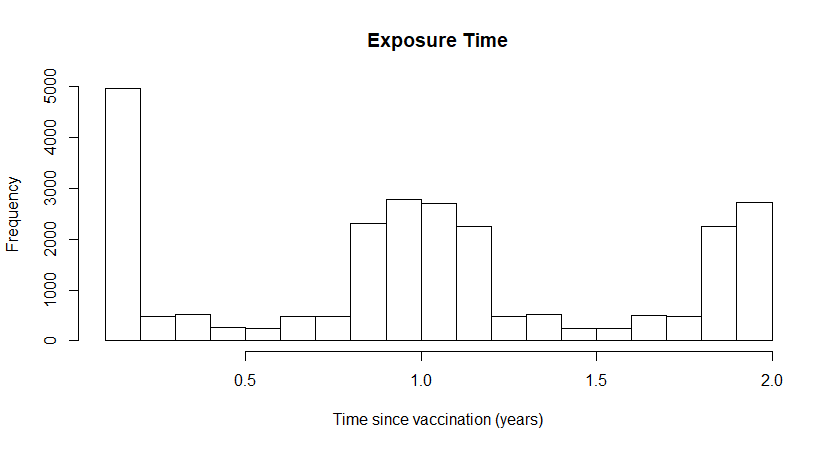


Figure S6 The overall proportion of infected cases detected in simulated trials using seroincidence as the primary outcome


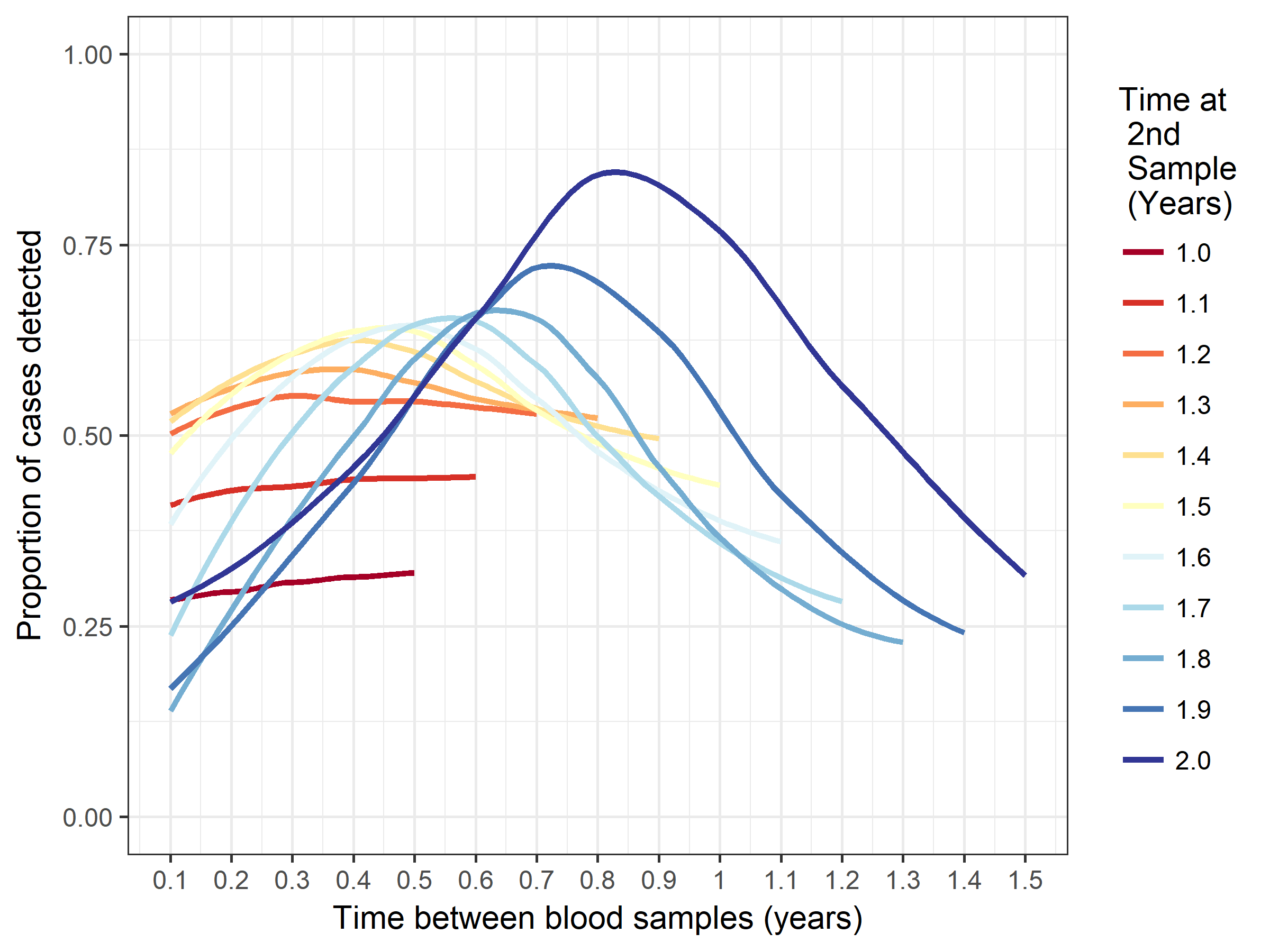


Figure S7 Bias in estimated relative risks in seroincidence studies


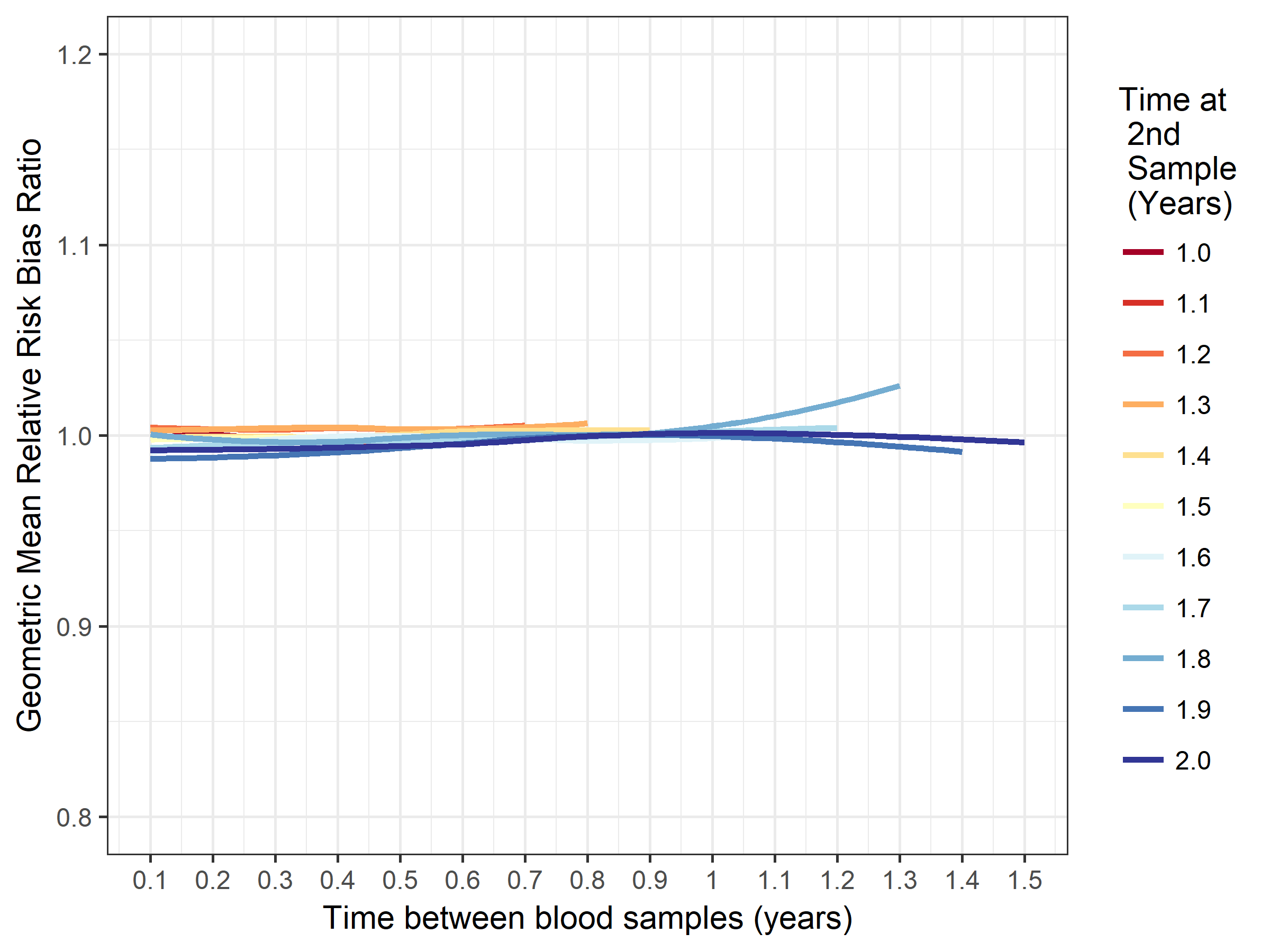


# R Code

#simulate typhoid clinical trials

#RR=1.0

require(ggplot2)

require(dplyr)

require(mixtools)

require(doBy)

library(plyr)

require(RColorBrewer)

#Vi-IgG after TCV (GMC 1000, log10 scale)

tcv.mu<-3.0

tcv.sd<-0.33

#set participant-specific parameters

n.participants<-50000

#list of participant ids

n<-seq(1,n.participants,1)

#antibody level one month after vaccination (starting point of curve)

intercept<-rnorm(length(n), mean=tcv.mu, sd=tcv.sd) # simulated antibody level 42 days post-vacc

d1<-as.data.frame(cbind(n,intercept))

names(d1)<-c("pat","intercept")

#paticipant-specific parameters for cubic polynomial decay

d1$poly1<-rnorm(length(d1$pat),mean=2.9,sd=0.1)

d1$poly2<-rnorm(length(d1$pat),mean=1.85,sd=0.02)

d1$poly3<-rnorm(length(d1$pat),mean=-0.4,sd=0.01)

#probability of exposure----------------------------------------------

pe<-0.5

d1$exposed<-rbinom(length(d1$pat),1,pe)

#set time of exposure------------------------------------------

t<-runif(n.participants, min=0.1, max=2.0)

t<-floor(t*10)/10

d1$t.exp<-t

#time since vacination--------------------------------------

time<-rep(seq(0,2,0.1),100) #21 timepoints x 100 participants

pat<-rep(n,21)

pat<-sort(pat)

dat<-as.data.frame(cbind(pat,time))

dat1<-merge(d1,dat,by="pat")

#IgG decay formula - cubic polynomial------------------------------------------

dat1$igg1<-dat1$intercept-dat1$poly1*time+ dat1$poly2*(time^2)+dat1$poly3*(time^3)

#set probability of infection -------------------------------------------------

dat1$prob.inf.t<-exp(1.7638+(-0.9597*dat1$igg1))/(1+exp(1.7638+(-0.9597*dat1$igg1)))

dat1$p.inf<-ifelse(round(dat1$time, digits=1)==round(dat1$t.exp,digits=1),dat1$prob.inf.t,NA)

p.inf<-dat1[!is.na(dat1$p.inf),10] #n=50000

d1<-cbind(d1,p.inf)

d1$infected<-rbinom(length(d1$pat),1,p.inf)

d1$infected[d1$exposed==0]<-0

overall<-prop.table(table(d1$infected)) #27.7% infected

denom<-overall[2]

#create second dataset of post-infection IgG decay (igg2)-------------------

intercept<-rnorm(length(n), mean=tcv.mu*.75, sd=tcv.sd*1.2) # simulated antibody level after exposure - smaller than the response to vaccination

d2<-as.data.frame(cbind(n,intercept))

names(d2)<-c("pat","intercept")

#paticipant-specific parameters for cubic polynomial decay

d2$poly1<-rnorm(length(d1$pat),mean=2.9,sd=0.1)

d2$poly2<-rnorm(length(d1$pat),mean=1.85,sd=0.02)

d2$poly3<-rnorm(length(d1$pat),mean=-0.4,sd=0.01)

#time since vacination------------------------------------------------------

dat2<-merge(d2,dat1[,c(1,7,8)],by="pat")

dat2$igg2<-dat2$intercept-dat2$poly1*time+ dat2$poly2*(time^2)+dat2$poly3*(time^3)

#add the $time.infect variable to each time in offset dataset------------------

dat2$time<-dat2$time+dat2$t.exp

#merge with original dataset by pat id and time-------------------------------

dat3<-merge(dat1,dat2,by=c("pat","time"), all=T)

dat3<-dat3[,names(dat3) %in% c("pat","time","igg1","igg2")]

dat3<-dat3[dat3$time<=2,]

dat3<-merge(dat3,d1,by="pat")

#replace igg1 with igg.infect if not NA--- only for those infected------------------------------------

dat3$igg<-dat3$igg1

dat3$replace<-ifelse((is.na(dat3$igg2)),dat3$igg1,log10(10^dat3$igg2+10^dat3$igg1))

#add the antibody on

dat3$igg<-ifelse(dat3$infected==1,dat3$replace,dat3$igg1)

#sample from the undelying population to create clinical trial dataset and estimate seroincidence--------------

#select participants in trial----------------------

set.seed(123456)

run.many<-function(n.reps){

allresults<-numeric(0)

output1<-(0)

allcomp1<-(0)

allcomp2<-(0)

for (i in 1:n.reps) {

select<-sample_n(d1, trial_size) #select 500 obs

pat<-select[,1] #pat ids for the selected participants

# randomly allocate to two groups with prob=0.5

randlist<-as.data.frame(cbind(pat,rbinom(length(pat),1,0.5)))

names(randlist)[2]<-"trt"

sub1a<-dat3[dat3$pat %in% pat, names(dat3) %in% c("pat","time","igg", "infected","intercept")]

#keep only time points for blood sampling and required vars

sub1<-merge(sub1a,randlist,by="pat")

time0<-sub1[sub1$time==0,]

props<-as.data.frame(prop.table(table(time0$infected,time0$trt),2))

samplerr<-props$Freq[2]/props$Freq[4]

#reshape data -

sub2<-reshape(sub1, direction="wide",

v.names="igg",

timevar="time",

idvar = "pat")

for (j in seq(25,15,-1)){ #2 years time point to 1 years time point - second blood sample

for (k in seq(10,(j-1),1)){ #1st blood sample - from 6 months to the time point 1 month before the second sample

#calculate differences between time points for each participant-------------

diff<-sub2[,j]-sub2[,k]

#fit mixture model to differences --------------------------------------

#2-component mixture model

mixmdl <- normalmixEM(diff)

#merge posterior probabilities back with the original ids and trt groups

out1<-cbind(randlist,mixmdl$posterior,mixmdl$x)

#ratio of the two summed probabilities (relative risk)

n1<-trial_size/2

n2<-n1

#which component is smallest?

comp<-ifelse(mixmdl$lambda[1]<mixmdl$lambda[2],"comp.1","comp.2")

min<-ifelse(mixmdl$lambda[1]<mixmdl$lambda[2],mixmdl$lambda[1],mixmdl$lambda[2])

if(comp=="comp.1"){

x1<-sum(out1$comp.1[out1$trt==1])

x2<-sum(out1$comp.1[out1$trt==0])

rr<-x1/n1/(x2/n2)

logrr<-log(rr)

var.logrr<-(1/x1)-(1/n1)+(1/x2)-(1/n2)

se.logrr<-sqrt(var.logrr)

lclrr<-exp(logrr-1.96*se.logrr)

uclrr<-exp(logrr+1.96*se.logrr)

sig<-ifelse(lclrr<1 & uclrr>1, 0,1)

result1<-cbind(min, mixmdl$lambda[1],mixmdl$lambda[2],x1,x2,rr,lclrr,uclrr,sig, i, j,k, samplerr)

allcomp1<-rbind(result1,allcomp1)

}

else if(comp=="comp.2"){

x1<-sum(out1$comp.2[out1$trt==1])

x2<-sum(out1$comp.2[out1$trt==0])

rr<-x1/n1/(x2/n2)

logrr<-log(rr)

var.logrr<-(1/x1)-(1/n1)+(1/x2)-(1/n2)

se.logrr<-sqrt(var.logrr)

lclrr<-exp(logrr-1.96*se.logrr)

uclrr<-exp(logrr+1.96*se.logrr)

sig<-ifelse(lclrr<1 & uclrr>1, 0,1)

result2<-cbind(min, mixmdl$lambda[1],mixmdl$lambda[2],x1,x2,rr,lclrr,uclrr,sig, i,j,k, samplerr)

allcomp2<-rbind(result2,allcomp2)

}

allresults<-rbind(allcomp1,allcomp2)

}#end for j loop

} # end for k loop

}

return(allresults)

}

trial_size<-500

n.reps<-500

output1<-as.data.frame(run.many(n.reps))

output1<-output1[output1$V2>0,]

output1$comparison<-paste0(output1$j,"-",output1$k)

output1$rrbias<-output1$rr/output1$samplerr

#summarise across the 500 iterations for each combination(comparison)

output1a<-output1[output1$rr != Inf,]

output1a<-output1a[output1a$uclrr != Inf,]

output.summary3<-ddply(output1a, .(comparison), summarize,

med.rr=median(rr),

gmr=exp(mean(log(rr))),

max.rr=max(rr, na.rm = TRUE),min.rr=min(rr),

p025=quantile(rr,probs=0.025),p975=quantile(rr,probs=0.975),

p25=quantile(rr,probs=0.25),p75=quantile(rr,probs=0.75),

seroinc=mean(min),

sero.p025=quantile(min,probs=0.025),sero.p975=quantile(min,probs=0.975),

sero.p25=quantile(min,probs=0.25),sero.p75=quantile(min,probs=0.75),

meanbias=exp(mean(log(rrbias))),

bias.p025=quantile(rrbias,probs=0.025),bias.p975=quantile(rrbias,probs=0.975),

bias.p25=quantile(rrbias,probs=0.25),bias.p75=quantile(rrbias,probs=0.75),

k=mean(k),j=mean(j),

rr.var=var(rr, na.rm = TRUE))

output.summary3$j_k<-output.summary3$j-output.summary3$k

output.summary3$j<-as.factor(output.summary3$j)

output.summary3$sero.propor<-output.summary3$seroinc/denom

levels(output.summary3$j)<-c("1.0", "1.1", "1.2" ,"1.3" ,"1.4", "1.5", "1.6", "1.7", "1.8", "1.9" ,"2.0")

ggplot(output.summary3)+

geom_smooth(aes(y=sero.propor, x=j_k, colour=j), size=1, method="loess", se=FALSE)+

theme_bw()+

scale_x_continuous(breaks=seq(1,15,1), labels=seq(0.1,1.5,0.1))+

scale_y_continuous(limits=c(0,1))+

scale_color_brewer(palette="RdYlBu")+

labs(colour="Time at \n 2nd \n Sample\n (Years)", y="Proportion of cases detected", x="Time between blood samples (years)")

dev.off()
